# Deubiquitinase inhibitor degrasyn suppresses metastasis by targeting USP5‐WT1‐E‐cadherin signalling pathway in pancreatic ductal adenocarcinoma

**DOI:** 10.1111/jcmm.14813

**Published:** 2019-12-17

**Authors:** Jiajia Li, Haiying Li, Weijian Zhu, Bin Zhou, Jianchao Ying, Jiansheng Wu, Huxiang Zhang, Hongwei Sun, Shenmeng Gao

**Affiliations:** ^1^ Department of Gastroenterology the First Affiliated Hospital of Wenzhou Medical University Wenzhou Zhejiang Province China; ^2^ Laboratory of Internal Medicine the First Affiliated Hospital of Wenzhou Medical University Wenzhou Zhejiang Province China; ^3^ Pathology Department the First Affiliated Hospital of Wenzhou Medical University Wenzhou Zhejiang Province China; ^4^ Department of Hepatobiliary and Pancreatic Surgery the First Affiliated Hospital of Wenzhou Medical University Wenzhou Zhejiang Province China

**Keywords:** deubiquitinase inhibitor, pancreatic ductal adenocarcinoma, ubiquitin‐specific protease, Wilm's tumour‐1

## Abstract

Wilm's tumour‐1 (WT1) is overexpressed in pancreatic ductal adenocarcinoma (PDAC) and enhances metastasis. Deubiquitination stabilizes target proteins, and inhibiting deubiquitination facilitates the degradation of target proteins. However, whether inhibiting deubiquitination of WT1 facilitates its degradation and presents anti‐cancer ability in PDAC is unknown. Here, we found that deubiquitinase inhibitor degrasyn rapidly induced the degradation of endogenous and exogenous WT1 through enhancing ubiquitination of WT1 followed by the up‐regulation of E‐cadherin. Knockdown of *WT1* by short hairpin RNAs (shRNAs) inhibited metastasis and overexpression of WT1 partially prevented degrasyn‐induced anti‐metastasis activity, suggesting that degrasyn presents anti‐metastasis activity partially through degrading WT1 protein. We further identified that USP5 deubiquitinated WT1 and stabilized its expression. The higher expressions of USP5 and WT1 are associated with tumour metastasis. More importantly, degrasyn inhibited the activity of USP5 and overexpression of USP5 partially prevented degrasyn‐induced degradation of WT1 protein, suggesting that degrasyn degraded WT1 protein through inhibiting the activity of USP5. Finally, degrasyn reduced the tumorigenicity in a xenograft mouse model and reduced the metastasis in vivo. Our results indicate that degrasyn presents strong anti‐cancer activity through USP5‐WT1‐E‐cadherin signalling in PDAC. Therefore, degrasyn holds promise as cancer therapeutic agent in PDAC with high expressions of USP5 and WT1.

## 1 INTRODUCTION

Pancreatic ductal adenocarcinoma cell (PDAC) is a deadly disease, because its overall 5‐year survival rate is only 6%.^1^ Although PDAC stands as the fourth leading cause of cancer‐related death at present, it will become second leading cause of cancer‐related death by the year 2030 or even earlier due to continuous increase in its incidence and mortality.^2^ PDAC is always found at late stages because of no obvious clinical features at early stages. Furthermore, unfortunately, most of the PDAC patients are not sensitive for almost all kinds of conventional chemotherapy and radiotherapy.^3^ Multiple research indicates that four major signature mutations, including in GTPase KRas (KRAS) oncogene, cyclin‐dependent kinase inhibitor 2A, SMAD family member 4 and p53 protein, facilitate pancreatic intraepithelial neoplasia lesions to develop into PDAC.^4^ Significant progresses have been made in the diagnosis and clinical therapy in PDAC in recent years, but undesirable side effects to normal tissues and drug resistance severely limit the application. Therefore, understanding pancreatic pathogenesis and developing effective strategies to treat PDAC are urgently needed.

The *Wilms tumour‐1* (*WT1*) gene located on human chromosome 11 p13 is firstly isolated and identified in Wilms' tumour, a childhood tumour of the kidney.^5^ WT1 encodes a 49‐52 kDa protein with four zinc fingers in C‐terminal domain and is important for normal urogenital development and cancer pathogenesis. Although *WT1* is firstly identified as tumour suppressor gene in Wilms' tumour, emerging data indicate that *WT1* acts as an oncogene and high expression of WT1 is frequently found in different types of cancers including pancreatic cancer,^6^ lung cancer^7^ and haematological malignancies.^8^ As transcript factor, WT1 enhances proliferation, inhibits apoptosis and suppresses differentiation through modulating several important genes, such as *Cyclin D1* 9 and *Bcl‐2*.^10^ More importantly, WT1 inhibits the expression of E‐cadherin through directly binding its promoter to enhance the metastasis.^7^, ^11^ All these reports suggest that ectopic expression of WT1 contributes to the pathogenesis of cancer and provides a potential candidate target for therapy. Therefore, inhibition of *WT1* gene by siRNA or degradation of WT1 protein by small molecular compounds such as curcumin^12^ presents anti‐cancer activity in pancreatic cancer.

Metastasis is the leading reason for the resultant mortality in more than 90% of cancer patients, including PDAC. Metastasis is a complex process in which the metastatic potential of PDAC cells is influenced by cell‐intrinsic identities and extrinsic microenvironment factors. E‐cadherin is an important marker of epithelial cells. The decreased E‐cadherin expression promotes the metastasis during early carcinogenesis progression.^13^ The expression of E‐cadherin is complicatedly regulated by many transcript factors, such as ZEB1/2 and Snail, which are induced by multiple signalling pathways including Wnt, TGF‐β and Notch.^14^ More importantly, E‐cadherin is negatively regulated by WT1.^9^ Thus, WT1‐E‐cadherin signalling pathway facilitates the metastasis in cancer cells.

Ubiquitin‐specific protease (USP) reverses protein ubiquitination and primarily counterbalances ubiquitin‐protein conjugation. USP contributes to the cleavage of ubiquitin from its precursors and unanchored polyubiquitin chains. Thus, inhibition of deubiquitinase contributes to the degradation of target oncoprotein.^15^, ^16^ Degrasyn is a small molecule compound initially identified as an inhibitor for Janus‐activated kinase (JAK)/signal transducer and activator of transcription (STAT) signalling pathway. Unlike AG490,^17^ degrasyn acts as a cell‐permeable USP inhibitor, leading to a rapid accumulation of protein‐ubiquitin conjugates and the formation of aggresomes.^18^ Degrasyn has been reported to present anti‐leukaemia activity through ubiquitin‐mediated degradation of c‐Myc^19^ and BCR‐ABL.^20^ However, whether degrasyn has anti‐cancer activity in PDAC through degradation of WT1 oncoprotein by inhibition of deubiquitination is largely unknown.

Here, we found that deubiquitinase inhibitor degrasyn presents a strong anti‐metastasis ability through USP5‐mediated down‐regulation of WT1 and up‐regulation of E‐cadherin in PDAC. More importantly, USP5, a ubiquitin‐specific protease, deubiquitinates WT1 protein and stabilizes its expression. Therefore, degrasyn presents anti‐metastasis ability through USP5‐WT1‐E‐cadherin axis and might be a lead compound for novel therapeutics of PDAC patients.

## 2 MATERIAL AND METHODS

### 2.1 Cell lines, tissue specimens and reagents

Pancreatic cancer cell lines (PANC‐1, BxPC‐3, AsPC‐1 and Capan‐1) and HDPE6C7 immortalized pancreatic duct epithelial cells (Chinese Academy of Sciences Cell Bank) were used in this study. All pancreatic cancer cells were cultured in either DMEM or RPMI‐1640 medium supplemented with 10% foetal bovine serum (FBS; Invitrogen) and cultured in a humidified 37°C incubator with 5% CO_2_. Surgical resection from pancreatic cancer specimens were performed from PDAC patients in the First Affiliated Hospital of Wenzhou Medical University. All the samples were stored in formalin for pathology analysis. Histological types of these patients were further analysed by an experienced pathologist using standard haematoxylin and eosin staining. Clinicopathological characteristics of the pancreatic cancer patients were shown in Table S1. Informed consents were obtained from all patients. This study was approved by the Research Ethnics Committee of the First Affiliated Hospital of Wenzhou Medical University. Proteasome inhibitor MG132 (Calbiochem), cycloheximide (CHX; Sigma‐Aldrich) and degrasyn (Selleckchem) were dissolved in dimethyl sulfoxide (DMSO). All these compounds were kept at −20°C until use.

### 2.2 Transwell migration and invasion assay

The migration and invasive abilities of PANC‐1 and BxPC‐3 were performed using Transwell (Corning Costar Corp). For migration assay, pancreatic cancer cells (5 × 10^4^) were put into the upper noncoated membrane (24‐well insert; pore size, 8 μm). For invasion assay, matrigel was firstly diluted with serum‐free culture medium and coated on membrane. Then, pancreatic cancer cells (1 × 10^5^) were put into the upper compartment per well with the Matrigel‐coated membrane (BD Biosciences). In both assays, pancreatic cancer cells were suspended in 200 μl RPMI 1640 containing 2% foetal bovine serum and were put into the upper compartment after 400 μl of RPMI 1640 containing 10% foetal bovine serum was added to the lower compartment. After incubation for 24 h at 37°C, the membrane inserts were removed from the plate, and non‐invading cells were removed from the upper surface of the membrane. Cells moving to the bottom surface of the chamber were fixed with 2% paraformaldehyde for 10 min and were stained with 0.1% crystal violet for 60 min. Finally, the cells were imaged and counted in at least 10 random fields by a CKX41 inverted microscope (Olympus).

### 2.3 Wound‐healing assay

Pancreatic cells were plated in 6‐well plates at 2.0 × 10^5^ cells/well. When cells reached 80% confluence, the individual wells were wounded by scratching with a pipette tip and incubated with medium containing with 10% FBS for 12 hours. The width of the scratch gap was viewed under the phase‐contrast microscope in four separate areas. Cells were photographed under microscopy, and the distance of cell motility was calculated.

### 2.4 Colony formation assay

Pancreatic cancer cells were seeded into 6‐well plates at a density of 2,000 cells per well and cultured for 10 days until visible clones appeared. Cell colonies were stained using the Giemsa solution and counted under a microscope. Three independent experiments were performed in quadruplicate.

### 2.5 Cell counting kit‐8 (CCK‐8 assay)

Pancreatic cancer cells were seeded into 96‐well plates (1.0 × 10^4^ cells/well) and allowed to attach overnight. After cellular adhesion, different concentrations of degrasyn were added in liquid supernatant. Cell viability was assessed by CCK‐8 assay (Dojin Laboratories). The absorbance at 450 nm (A450) of each well was read on a spectrophotometer. Three independent experiments were performed in quadruplicate.

### 2.6 Immunohistochemical staining (IHC)

Paraformaldehyde‐fixed paraffin‐embedded sections of human tumour tissues were subjected to IHC following by standard protocols. The following antibodies were used: anti‐USP5 (1:200, ab244290; Abcam) and anti‐WT1 (1:200, ab89901; Abcam). To determine the relative expressions of USP5 and WT1, the integrated optical densities of USP5 (Table S1) and WT1 (Table S2) by IHC were analysed with Image Pro Plus 6.0 (Media Cybernetics) and representative pictures were captured by Leica DM4000B microscope (Leica).

### 2.7 Western blot

Western blot analysis was performed using standard techniques. Briefly, pancreatic cancer cells were digested and lysed by RIPA buffer supplemented with sodium orthovanadate, phenylmethylsulfonyl fluoride (PMSF), protease and phosphatase inhibitors (Thermo Scientific). Protein concentration was determined by a BCA assay (Pierce). All lysates were separated by SDS‐PAGE, and then, the separated proteins were transferred to nitrocellulose membrane (Thermo Scientific). The membranes were blocked with 5% bovine serum albumin in PBS with 0.1% Tween 20 at 37°C for 2 h. The transferred membrane was blotted with the following primary antibodies: WT1 (1:5000, ab89901; Abcam); E‐cadherin (1:2000, ab40772; Abcam); HSP90 (1:2000, ab13492; Abcam); USP5 (1:2000, ab155993; Abcam); Flag (1:1000, ab125243; Abcam); HA (1:1000, AE008; Abclone Biotechnology); β‐actin (1:5000, ab8227, Abcam). All secondary antibodies are conjugated with horseradish peroxidase (HRP). Signals were detected by chemiluminescence reagents (Thermo Scientific).

### 2.8 Plasmid construction

To produce the vector overexpressing WT1, the whole coding sequence (CDS) of WT1 (isoform A, NM_000378; Ex5‐KTS‐) was cloned into pCMV‐3Tag‐1 (Stratagene) and murine stem cell virus pMSCV‐puro (Clontech).^8^ WT1 deletion at amino acids 374 to 398 (Zn1), 404 to 428 (Zn2), 434 to 456 (Zn3) and 462 to 486 (Zn4) were constructed on pCMV‐WT1 by using the QuickChange site‐directed mutagenesis kit (Stratagene) according to the manufacturer's instruction. pCDNA‐HA‐ubiquitin (HA‐Ub) was purchased from Addgene. The coding sequence of USP5 was constructed in lentivirus vector pLVX‐puro (Clontech) to overexpress USP5. The primer sequences for construction of plasmids were indicated in Table S3. All the plasmids were confirmed by direct DNA sequence.

### 2.9 Other procedures

For details on RNA interference, retrovirus and lentivirus production and cell transduction, RNA extraction and qRT‐PCR, co‐immunoprecipitation (co‐IP) assay, in vitro deubiquitination assay and other experiments see Supplemental Materials and Methods.

### 2.10 Statistical analysis

All the results were expressed as Mean ± *SD* where applicable. The significance of the difference between groups was determined by Student's t test. A *P* value of <.05 was considered statistically significant. The Mann‐Whitney *U* test was applied to analyse the correlation between the integrated optical densities of USP5, WT1 and clinicopathologic characteristics. All statistical analyses were performed with SPSS software (SPSS 22.0).

## 3 RESULTS

### 3.1 Anti‐cancer activity of degrasyn in pancreatic cancer cells

**Figure 1:**
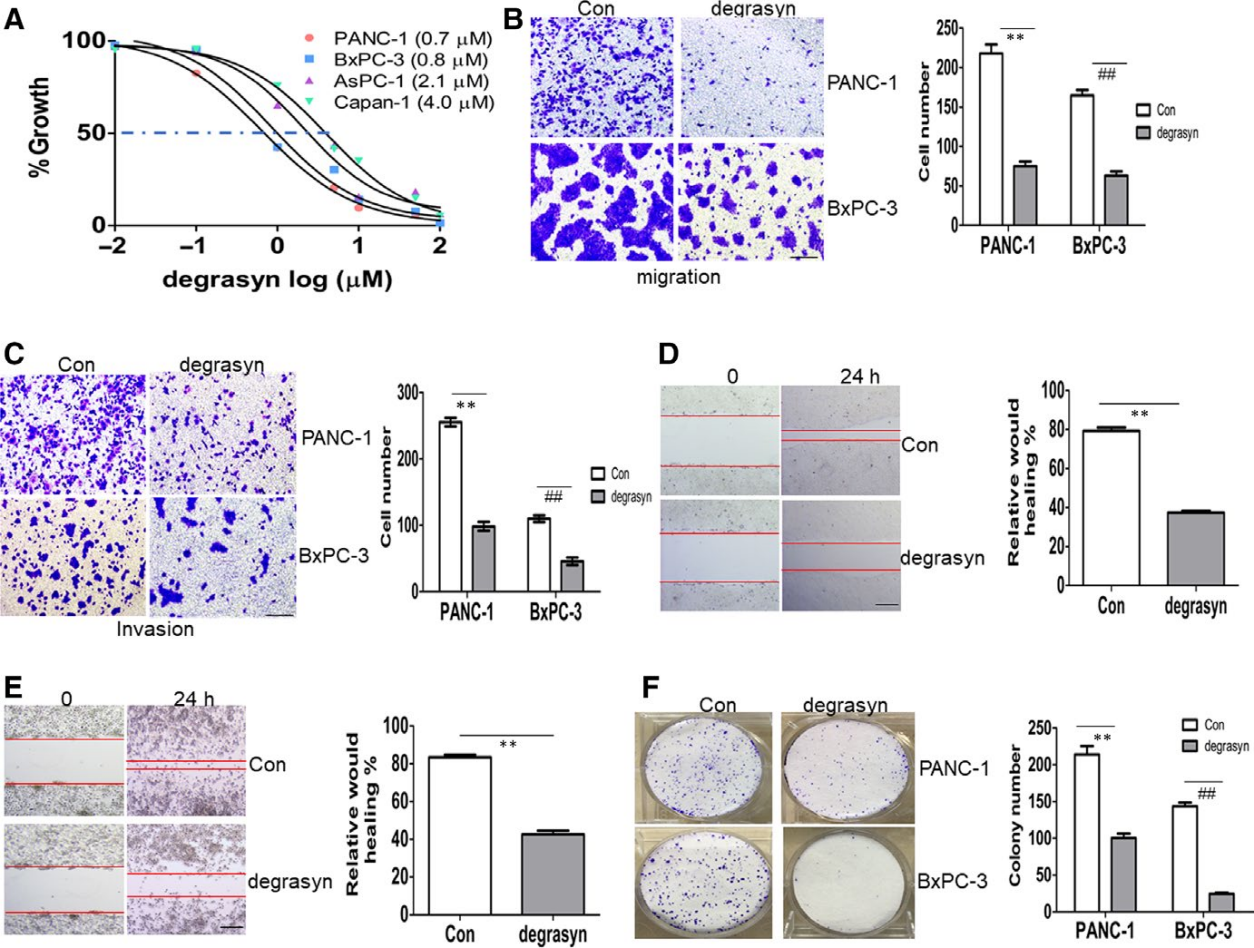
The anti‐cancer ability of degrasyn in pancreatic cancer cells. (A) Four pancreatic cancer cell lines were treated with different concentrations of degrasyn for 24 hours. Cell growth was measured by CCK‐8 assay. A 50% inhibitory concentration (IC50) of degrasyn was calculated for the four pancreatic cancer cell lines. (B and C) Transwell migration (B) and invasion (C) assays were performed in PANC‐1 and BxPC‐3 cells treated with or without 1 μM degrasyn for 24 hours. ** and ^##^ *P* < .01. Shown is the representative plot (Left) and summary of cell number (Right). (D and E) Wound‐healing assay was performed in PANC‐1 (D) and BxPC‐3 cells (E) treated with or without 1 μM degrasyn. ** and ^##^ *P* < .01. Shown is the representative picture (Left) and summary of relative would healing (Right). (F) Colony formation was performed by methylene blue staining in PANC‐1 and BxPC‐3 cells treated with or without 1 μM degrasyn. ** and ^##^ *P* < .01. Shown is the representative plot (Left) and summary of colony number (Right)

To investigate whether deubiquitinase inhibitor degrasyn has anti‐cancer activity in PDAC, 50% inhibition of cell growth (IC50) values were calculated in four pancreatic cancer cell lines, which were treated with different concentrations of degrasyn for 24 h. IC50 values were among 1‐5 μM in four pancreatic cancer cell lines (Figure 1A). PANC‐1 and BxPC‐3 cells were finally selected for the next experiments because they presented more sensitive anti‐growth activity of degrasyn than did Aspc‐1 and Capan‐1 cells. For this purpose, migration and invasion were firstly evaluated in PANC‐1 and BxPC‐3 cells treated with degrasyn (1 μM). Degrasyn substantially reduced the migration (Figure 1B) and invasion (Figure 1C) in PANC‐1 and BxPC‐3 cells. Furthermore, wound‐healing test representing cell motility was assessed in pancreatic cancer cells. Also, degrasyn remarkedly reduced above half of the cell motility of pancreatic cancer cells (Figure 1D,E). Finally, colony formation was performed. Degrasyn decreased above half of the colony in PANC‐1 cells and almost completely inhibited colony formation in BxPC‐3 cells (Figure 1F). All these data demonstrate that degrasyn presents strong anti‐metastasis in pancreatic cancer cells.

### 3.2 Degrasyn reduces WT1 protein level by enhancing the ubiquitination of WT1 and zinc finger region is required for degrasyn‐induced degradation of WT1 protein

**Figure 2:**
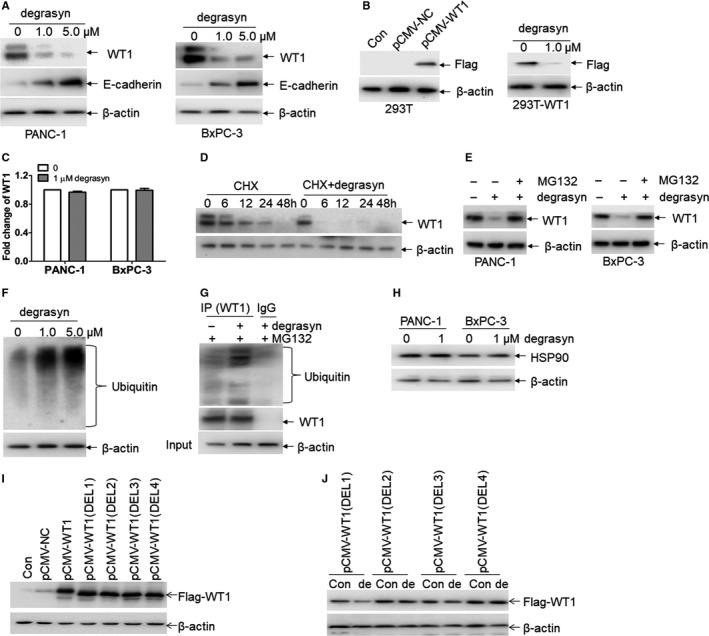
Degrasyn induces the degradation of WT1 protein through enhancing ubiquitination. (A) The protein expressions of WT1 and E‐cadherin were measured in PANC‐1 and BxPC‐3 cells treated with or without 1 μM and 5 μM degrasyn for 24 hours. (B) Flag tag (Flag‐WT1) was detected in 293T cells transfected with pCMV‐NC or pCMV‐WT1 (Left blots). Flag tag was detected in 293T‐WT1 cells incubated with or without 1 μM degrasyn for 24 hours (Right blots). (C) The transcriptional expressions of *WT1* were measured in PANC‐1 and BxPC‐3 cells treated with or without 1 μM degrasyn for 24 hours. (D) WT1 protein was detected in 10 μg/ml cycloheximide (CHX)‐treated PANC‐1 and BxPC‐3 cells, which were incubated with or without 1.0 μM degrasyn for the indicated times. (E) The protein expression of WT1 was measured in PANC‐1 and BxPC‐3 cells, which were treated with 1.0 μM degrasyn for 24 hours in the presence or absence of 5 μM MG132. (F) Ubiquitin was detected in PANC‐1 cells treated with 1.0 and 5.0 μM degrasyn for 4 hours. (G) PANC‐1 cells were treated with or without degrasyn, followed by the co‐immunoprecipitation (co‐IP) with anti‐WT1 antibody or normal mouse IgG. MG‐132 (5 μM) was added 4 hours before cell harvest. The Western blots were taken for ubiquitin and WT1 in co‐IP complex and input by anti‐β‐actin, respectively. (H) The protein expression of HSP90 was measured in PANC‐1 and BxPC‐3 cells, which were treated with or without degrasyn. (I) Flag tag (Flag‐WT1) was detected in 293T cells transfected with pCMV‐NC, wide‐type pCMV‐WT1 and four different pCMV‐WT1 deletions of zinc finger region. (J) 293T cells were transfected with four pCMV‐WT1 deletions for 24 hours and then treated with 1.0 μM WP1130 for 24 hours. Flag taq was measured by Western blot

As reported, degrasyn induces the degradation of target proteins through inhibiting deubiquitinase activity.^18^ WT1 is an important oncogene and is highly overexpressed in PDAC than in normal pancreatic tissues.^12^ We then determined whether degrasyn induces the degradation of WT1 protein through enhancing ubiquitination of WT1. PANC‐1 and BxPC‐3 cells were treated with 1.0 μM and 5.0 μM degrasyn for 24 hours, followed by Western blot for endogenous WT1 protein. Degrasyn (1 μM and 5.0 μM) almost completely degraded WT1 protein in pancreatic cancer cell lines (Figure 2A) and simultaneously increased the expression of E‐cadherin (Figure 2A), which has been reported to be negatively regulated by WT1.^21^, ^22^ To explore whether degrasyn degrades the exogenous WT1 protein, 293T cells were transfected with pCMV‐WT1 overexpressing Flag‐WT1. As expected, degrasyn rapidly degraded exogenous WT1 protein by measuring Flag expression (Figure 2B). To determine whether degrasyn modulates transcriptional expression of *WT1*, qRT‐PCR was performed to measure transcriptional expression of *WT1* in pancreatic cancer cells treated with degrasyn. However, degrasyn did not affect the transcriptional expression of *WT1* (Figure 2C).

Considering that degrasyn decreases WT1 protein expression but not affects mRNA level of *WT1*, we asked whether degrasyn degrades WT1 protein through post‐transcriptional manner. For this purpose, PANC‐1 cells were treated with protein synthesis inhibitor cycloheximide (CHX) with or without degrasyn for different times. The half‐time of WT1 protein in degrasyn‐treated cells was obviously shortened than that in untreated cells (Figure 2D). These results suggest that degrasyn treatment reduces the stability of WT1 protein.

As reported, most proteins are degraded through ubiquitin‐proteasome pathway.^23^ To explore whether degrasyn degrades WT1 protein through ubiquitin‐proteasome pathway, degrasyn‐treated pancreatic cancer cells were incubated with or without MG132, an inhibitor for ubiquitin‐proteasome pathway. Degrasyn‐induced degradation of WT1 in PANC‐1 and BxPC‐3 cells was almost completely blocked by MG132 (Figure 2E), indicating that degrasyn‐induced degradation of WT1 is mediated by ubiquitin‐proteasome pathway. As reported, degrasyn enhances cellular protein ubiquitination via inhibiting deubiquitinases (DUB) activity.^18^ Consistent with it, degrasyn induced the ubiquitination accumulation in PANC‐1 cells (Figure 2F). To further confirm that degrasyn enhances ubiquitination of WT1 protein, co‐immunoprecipitation with anti‐WT1 protein and Western blot for ubiquitin were performed in pancreatic cancer cells. Degrasyn enhanced the expression of ubiquitinated WT1 protein (Figure 2G).

As reported, heat shock protein 90 (HSP90) regulates the expression of WT1 through associating with WT1 protein and stabilizes its expression. Furthermore, inhibiting HSP90 by 17‐AAG effectively decrease the expression of WT1 protein.^24^ We then asked whether degrasyn decreases WT1 protein through inhibiting HSP90. For this purpose, HSP90 protein expression was measured in degrasyn‐treated pancreatic cancer cells. Degrasyn did not modulate the protein expression of HSP90 in PANC‐1 and BxPC‐3 cells, suggesting that degrasyn‐induced degradation of WT1 protein is independent of HSP90 (Figure 2H).

Because the zinc finger region of WT1 is involved in DNA binding and protein‐protein interactions,^25^ we then determined whether zinc finger region is required for the degradation of WT1 protein by WP1130. For this purpose, four different zinc finger regions in wild‐type WT1 were deleted, in turn, and then were constructed in pCMV vector, respectively (Figure S1A). These pCMV‐WT1 (Del) vectors were transfected in 293T cells and Western blot by Flag antibody indicated the successful overexpression of WT1, which is deleted in zinc finger regions (Figure 2I). Finally, 293T cells were transfected with four different pCMV‐WT1 (Del) vectors, followed by the treatment with WP1130. As indicated in Figure 2J, degrasyn degraded pCMV‐WT1 (Del1) but not pCMV‐WT1 (Del2), pCMV‐WT1 (Del3) and pCMV‐WT1 (Del4), suggesting that zinc finger region 2, 3 and 4 are required for the degradation of WT1 protein by degrasyn.

### 3.3 Knockdown of WT1 suppresses proliferation and migration in pancreatic cells

**Figure 3:**
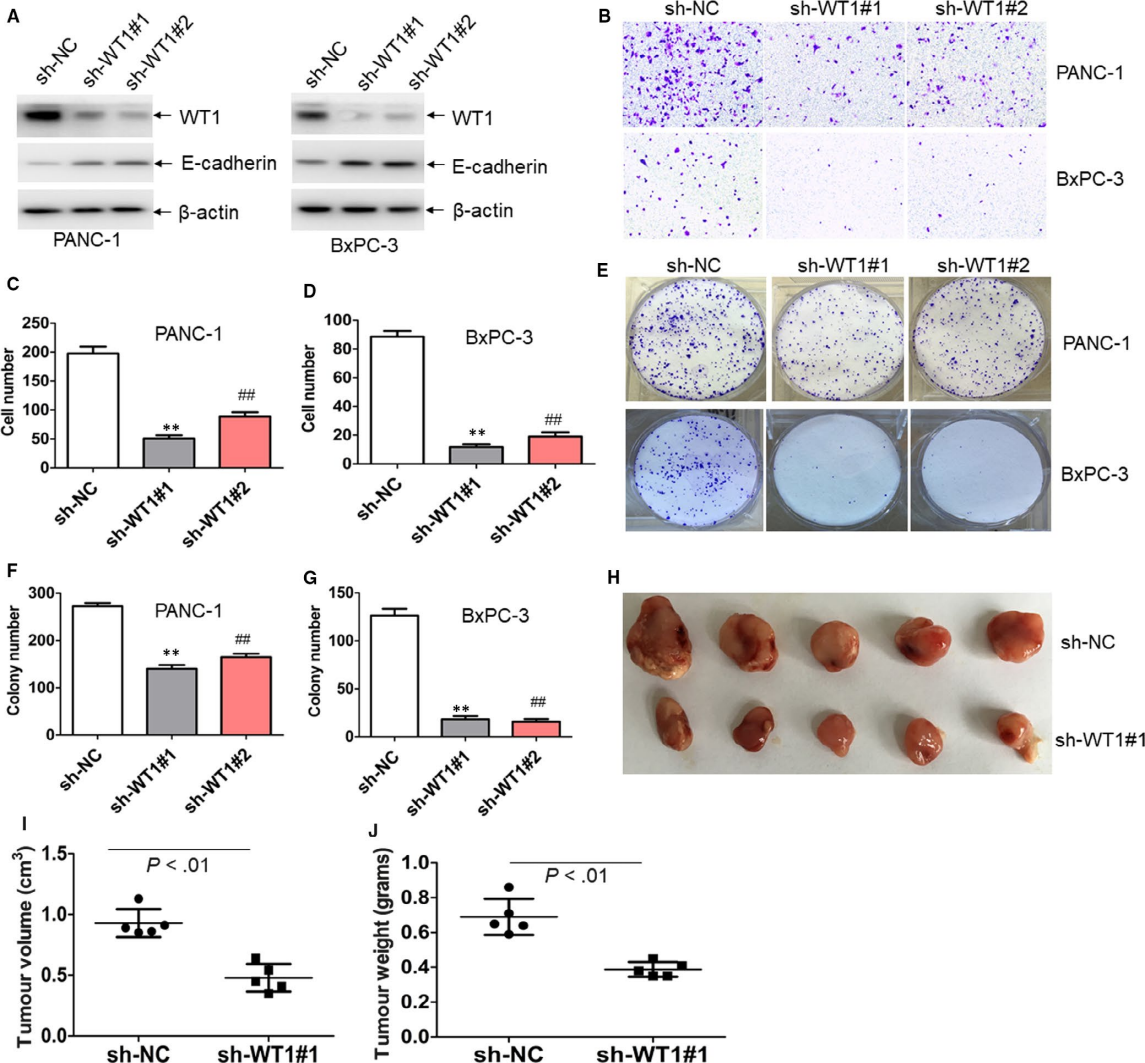
Knockdown of WT1 resembles the anti‐cancer activity of degrasyn. (A) The protein expressions of WT1 and E‐cadherin were detected in PANC‐1 and BxPC‐3 cells, which were transduced with two specific shRNAs for WT1 (sh‐WT1#1 and sh‐WT1#2) or non‐specific shRNA (sh‐NC) followed by puromycin selection for seven days. (B–D) Transwell migration assays were performed in PANC‐1 and BxPC‐3 cells transduced with sh‐WT1#1, sh‐WT1#2 or sh‐NC. ** and ^##^ *P* < .01. Shown is the representative plot (B) and summary of cell number (C and D). (E–G) Colony formation was performed in PANC‐1 and BxPC‐3 cells transduced with sh‐WT1#1, sh‐WT1#2 or sh‐NC. ** and ^##^ *P* < .01. Shown is the representative plot (E) and summary of colony number (F and G). (H–J) PANC‐1 cells with sh‐NC or sh‐WT#1 were injected subcutaneously into right flank of mice to construct xenograft mouse model. (H) A photograph of tumours in mice transplanted with PANC‐1 cells, which were transduced with sh‐NC (N = 5) or sh‐WT1#1 (N = 5). (I) Volumes of all tumours were measured when the experiment was terminated. (J) Net weights of all tumours were measured at the termination of the experiment

Having shown that degrasyn degrades WT1 protein and inhibits metastasis, we directly addressed the role of WT1 knockdown in the anti‐metastasis activity. To determine whether WT1 knockdown resembles the anti‐metastasis activity of degrasyn in our experimental model, pancreatic cancer cells were transduced with specific shRNAs for *WT1* (sh‐WT1#1 and sh‐WT1#2). As shown in Figure 3A, the protein levels of WT1 were substantially decreased by two specific shRNAs. Meanwhile, knockdown of WT1 significantly increased the expression of E‐cadherin in PANC‐1 and BxPC‐3 cells (Figure 3A). Accordingly, knockdown of WT1 reduced the migration (Figure 3B–D). Furthermore, WT1 knockdown substantially reduced colony formation in PANC‐1 and BxPC‐3 cells (Figure 3E–G).

To further explore the anti‐tumorigenicity by WT1 knockdown in pancreatic cancer cells, PANC‐1 cells with sh‐NC or sh‐WT#1 were injected subcutaneously into right flank of mice to construct xenograft mouse model. Tumours in mice transplanted with PANC‐1‐sh‐WT1#1 were significantly smaller than those in mice transplanted with PANC‐1‐sh‐NC. (Figure 3H). Furthermore, the average tumour volume in mice transplanted with PANC‐1‐sh‐WT1#1 reduced by 49% than that in control mice (Figure 3I). Also, the average tumour weight reduced 45% in mice transplanted with PANC‐1‐sh‐WT1#1 than that in control mice (Figure 3J).

### 3.4 Overexpression of WT1 partially prevents degrasyn‐induced anti‐cancer activity

**Figure 4:**
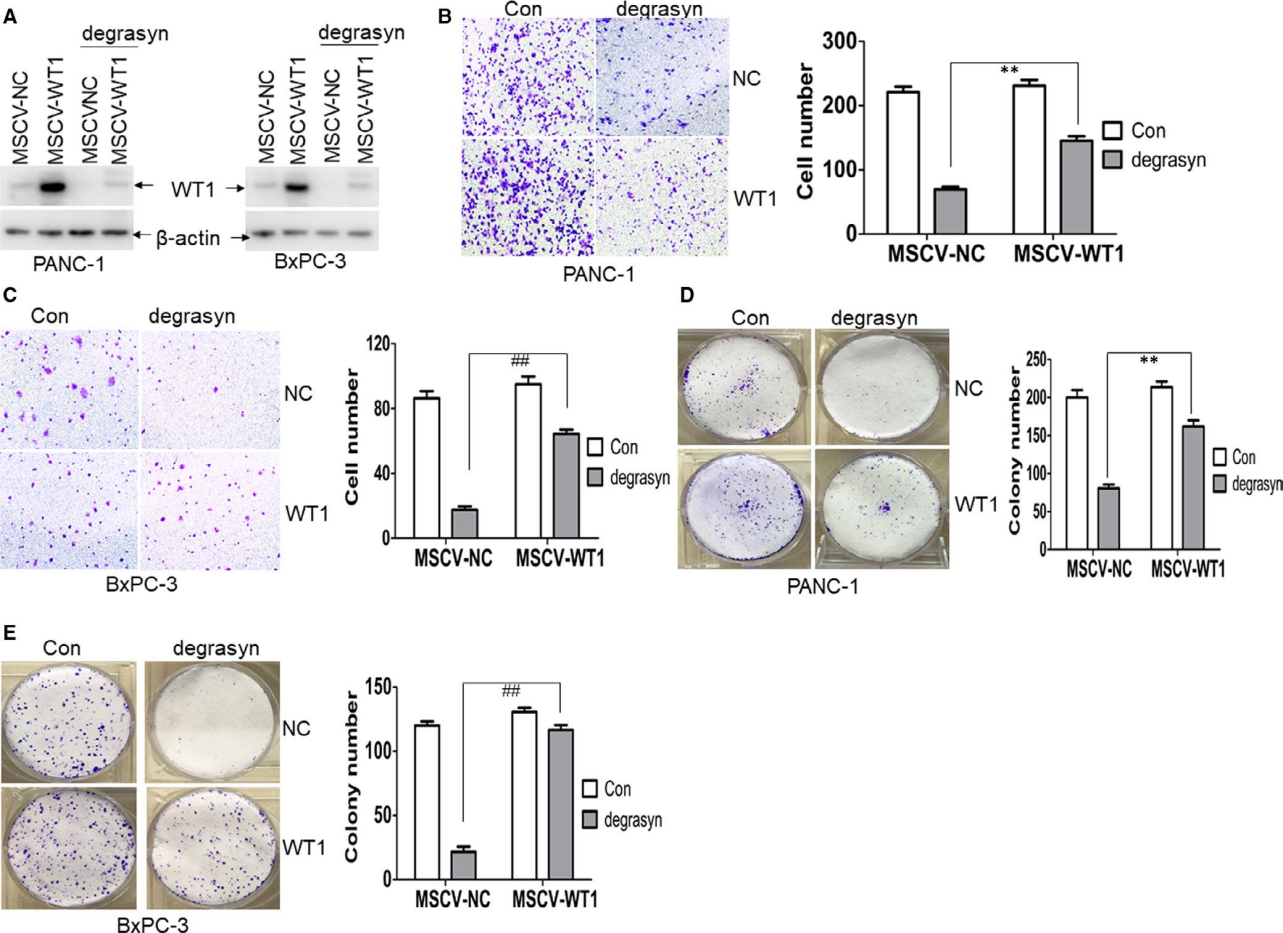
Overexpression of WT1 partially prevents degrasyn‐induced anti‐cancer activity. (A) PANC‐1 and BxPC‐3 cells were transduced with retroviral negative control (MSCV‐NC) or MSCV‐WT1, which overexpresses WT1. Positive clones were selected by puromycin for seven days. Transduced‐PANC‐1 and BxPC‐3 cells were treated with or without 1.0 μM degrasyn for 24 hours, and WT1 expression was detected by Western blot. (B and C) Transwell migration assays were performed in PANC‐1 (B) and BxPC‐3 cells (C), which were transduced with MSCV‐NC or MSCV‐WT1 and treated with or without 1.0 μM degrasyn for 24 hours. ** and ^##^ *P* < .01. (D and E) Colony formation was performed by methylene blue stain in PANC‐1 (D) and BxPC‐3 cells (E), which were transduced with MSCV‐NC or MSCV‐WT1 followed by 1.0 μM degrasyn treatment or not for 24 hours. ** and ^##^ *P* < .01

To determine whether degrasyn‐induced anti‐cancer activity depends on the degradation of WT1 protein, pancreatic cancer cells were transduced with MSCV‐WT1 or MSCV‐NC followed by the treatment of degrasyn. Successful overexpression of WT1 was detected by Western blot in PANC‐1 and BxPC‐3 cells (Figure 4A). We further measured the migration induced by the overexpression of WT1. Overexpression of WT1 slightly increased the cell migration than NC (Figure 4B,C). As expected, degrasyn‐induced inhibition of migration was in part prevented by the overexpression of WT1 than NC (Figure 4B,C). Finally, colony number was counted in degrasyn‐treated pancreatic cancer cells transduced with WT1 or NC. The decreased colony by degrasyn was substantially rescued by the overexpression of WT1 than NC (Figure 4D,E).

### 3.5 USP5 mediates degrasyn‐induced degradation of WT1 protein

**Figure 5:**
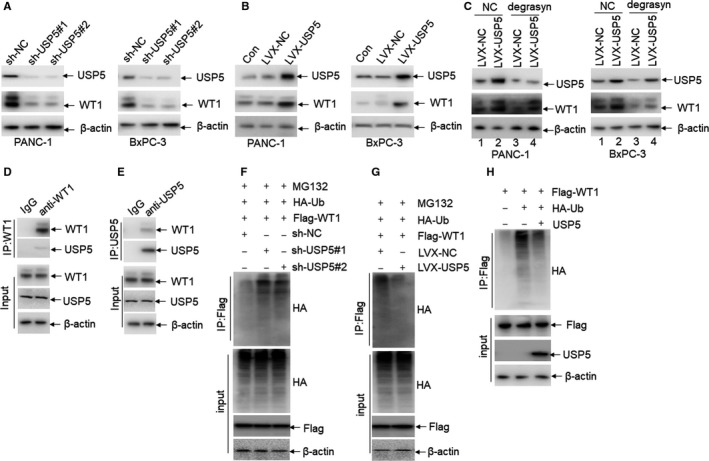
USP5 deubiquitinates WT1 protein. (A) The protein expressions of USP5 and WT1 were detected in PANC‐1 and BxPC‐3 cells, which were transduced with two specific shRNAs for USP5 (sh‐USP5#1 and sh‐USP5#2) or sh‐NC, followed by puromycin selection for seven days. (B) The protein expressions of USP5 and WT1 were detected in PANC‐1 and BxPC‐3 cells, which were transduced with LVX‐NC or LVX‐USP5 overexpressing USP5, followed by puromycin selection for seven days. (C) USP5 and WT1 expressions were detected in PANC‐1 and BxPC‐3 cells, which were transduced with LVX‐NC or LVX‐USP5 and treated with or without 1.0 μM degrasyn for 24 hours. (D) PANC‐1 lysates were immunoprecipitated by non‐specific IgG antibody and anti‐WT1 antibody, respectively. WT1 and USP5 were blotted in the immunoprecipitated lysates. (E) PANC‐1 lysates were immunoprecipitated by non‐specific IgG antibody and anti‐USP5, respectively. WT1 and USP5 were blotted in the immunoprecipitated lysates. (F) PANC‐1 cells were transduced with shRNAs for USP5 or sh‐NC together with HA‐Ub and Flag‐WT1. After treatment of MG132 (5 μM) for 4 hours, cell lysates were collected for IP assay with anti‐Flag antibody followed by Western blot with anti‐HA. (G) PANC‐1 cells were transduced with LVX‐USP5 or LVX‐NC together with HA‐Ub and Flag‐WT1, followed by MG132 (5 μM) treatment for 4 hours. Cellular extracts were prepared for IP assays with anti‐Flag followed by Western blot with anti‐HA antibody. (H) 293T cells were cotransfected with HA‐tagged ubiquitin and Flag‐WT1. Ubiquitinated‐WT1 protein was purified by anti‐Flag antibody and incubated with purified USP5 protein at 37 ℃ for 2 hours in vitro, followed by Western blot with anti‐HA antibody

As reported that degrasyn mainly inhibits the activity of USP5, USP9x and USP14,^18^ we then hypothesized that degrasyn degrades WT1 protein via inhibiting USP5, USP9x or USP14. The protein expression of WT1 was measured in pancreatic cancer cells, which were transduced with specific shRNAs for USP5, USP9x or USP14, respectively. Western blot indicates the successful knockdown of USP5 by two specific shRNAs and knockdown of USP5 significantly down‐regulated WT1 expression (Figure 5A). However, knockdown of USP9x and USP14 did not modulate the expression of WT1 (Figure S2A,B). To further confirm the deubiquitination ability of USP5 on WT1, PANC‐1 and BxPC‐3 were transduced with LVX‐USP5, which overexpresses USP5. Overexpression of USP5 substantially increased the expression of WT1 (Figure 5B). We then determined whether USP5 mediates degrasyn‐induced degradation of WT1 protein in pancreatic cancer cells. PANC‐1 and BxPC‐3 were transduced with LVX‐USP5 or LVX‐NC and then treated with degrasyn. As expected, degrasyn‐induced degradation of WT1 protein was partially rescued by the overexpression of USP5 (Figure 5C).

Considering that ubiquitin‐specific proteases interact with and deubiquitinate target proteins, we asked whether USP5 and WT1 interact with each other. For this purpose, the co‐immunoprecipitation (co‐IP) assays were performed in PANC‐1 cells. As shown in Figure 5D, the antibody for WT1 but not non‐related IgG successfully pulled down both WT1 and USP5. Meanwhile, the antibody for USP5 pulled down both USP5 and WT1 (Figure 5E). As a USP, USP5 stabilizes the expression of target protein via deubiquitinating target protein. For this purpose, the deubiquitination assay was performed in pancreatic cancer cells. Ubiquitin on WT1 protein was obviously increased in PDAC cells transfected with sh‐USP5 compared with NC (Figure 5F), while ubiquitin on WT1 protein was decreased in PDAC cells transfected with overexpression of USP5 compared with NC (Figure 5G).

To further confirm that USP5 directly deubiquitinates WT1 protein, we performed an in vitro deubiquitination assay. Ubiquitinated WT1 protein was purified from 293T cells and was incubated with commercially available purified USP5 protein. As demonstrated in Figure 5H, USP5 substantially decreased the ubiquitination of WT1. All these data suggest that WT1 might be a direct substrate for the deubiquitinase USP5.

### 3.6 High expression of USP5 is associated with tumour metastasis in PDAC

**Figure 6:**
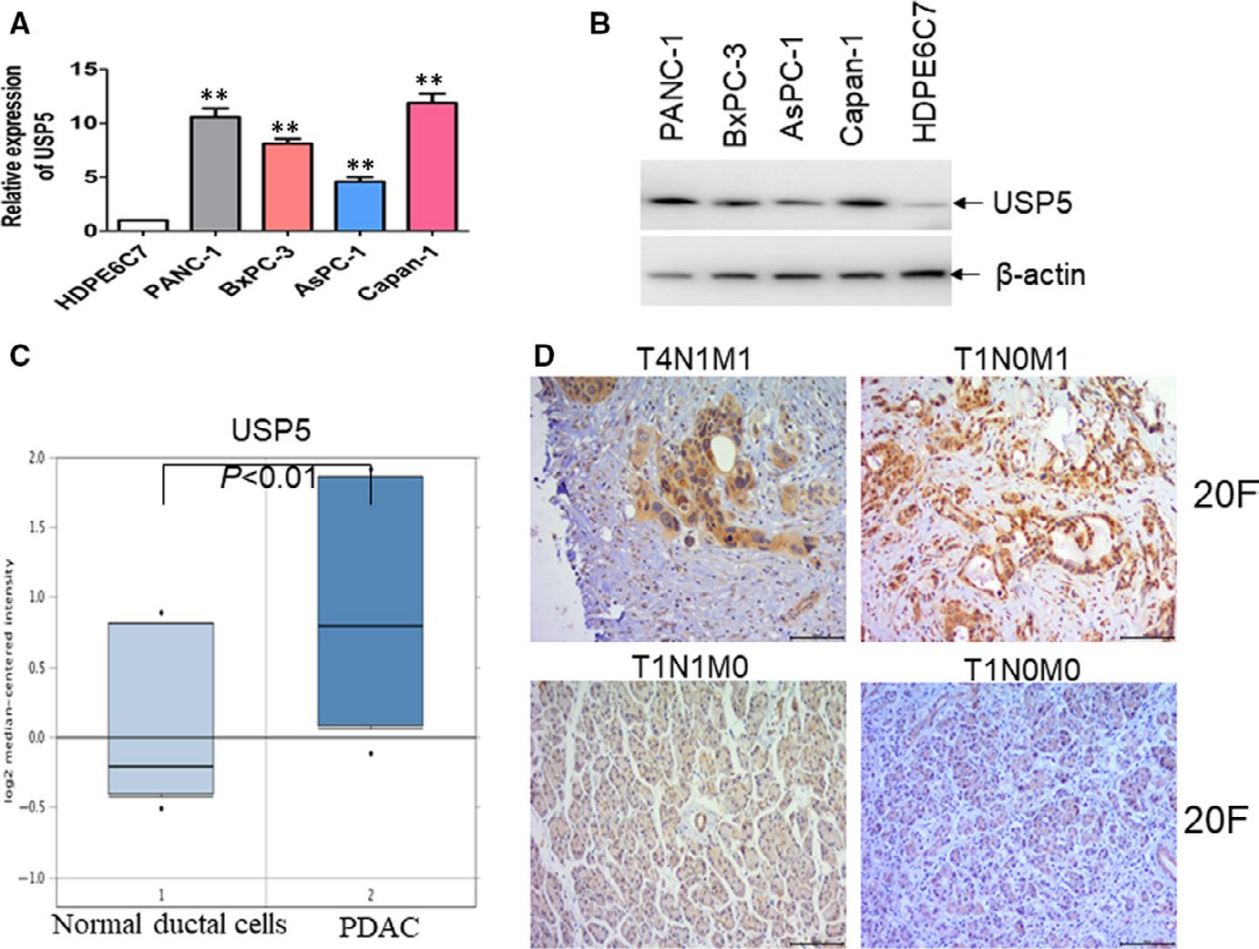
USP5 is associated with metastasis in PDAC patients. (A and B) The mRNA (A) and protein (B) expressions of USP5 were detected in four pancreatic cancer cell lines and HPDE6C7 cells. **P < .01 versus HPDE6C7 as normal control. (C) The mRNA expression of *USP5* was analysed in a database (https://www.ebi.ac.uk/arrayexpress/ experiments/E‐MEXP‐950). (D) Representative immunohistochemical staining for USP5 in PDAC tissues with different tumour stages

Because previous reports indicate that WT1 is overexpressed in PDAC tissues^6^, ^12^ and our studies indicate that USP5 stabilizes WT1 protein expression via deubiquitinating WT1 protein, we hypothesized that overexpression of USP5 in PDAC substantially increases the protein level of WT1. For this purpose, the mRNA and protein expressions of USP5 were firstly detected in four pancreatic cancer cell lines and HPDE 6C7, a duct epithelial cell lines as normal control. Both mRNA and protein expressions of USP5 were substantially increased in four pancreatic cancer cells than in HPDE 6C7 cells (Figure 6A,B). The mRNA expression of *USP5* was further analysed in 11 pancreatic ductal tissues and 11 ductal adenocarcinoma tissues in a database (https://www.ebi.ac.uk/arrayexpress/ experiments/E‐MEXP‐950). The mRNA expression of *USP5* was significantly up‐regulated in ductal adenocarcinoma tissues than in pancreatic ductal tissues (Figure 6C). We then determined whether USP5 protein expression by immunohistochemistry is associated with metastasis in clinical PDAC patients. To explore the expressions of USP5 in PDAC tissues, 46 cases of ductal adenocarcinoma at different tumour stages (Table S1) were used to detect the expression of USP5 by immunohistochemical staining. The expression of USP5 was associated with tumour metastasis (Figure 6D and Table S1). However, no obvious association was found between USP5 expression and sex, age, tumour differentiation, primary tumour or regional metastatic lymph nodes, respectively (Table S1).

To further confirm that WT1 is overexpressed in PDAC tissues than normal pancreatic tissues,^6^ we firstly analysed WT1 expression in Genotype‐Tissue Expression (GTEx) portal database^26^ and TCGA database. The expression of WT1 was increased in PDAC tissues compared with normal pancreatic tissues (Figure S3A). Then, we analysed the expression of WT1 in 46 cases of ductal adenocarcinoma by IHC staining. The expression of WT1 was associated with tumour metastasis (Figure S3B and Table S2). However, no significant association was found between WT1 expression and sex, age, primary tumour, tumour differentiation or regional metastatic lymph nodes, respectively (Table S2). Finally, we found that the expression of WT1 is positively associated with the expression of USP5 in PDAC tissues (*P* < .01, Figure S3C).

### 3.7 Degrasyn reduces tumorigenicity in a xenograft mouse model and reduces metastasis in vivo

**Figure 7:**
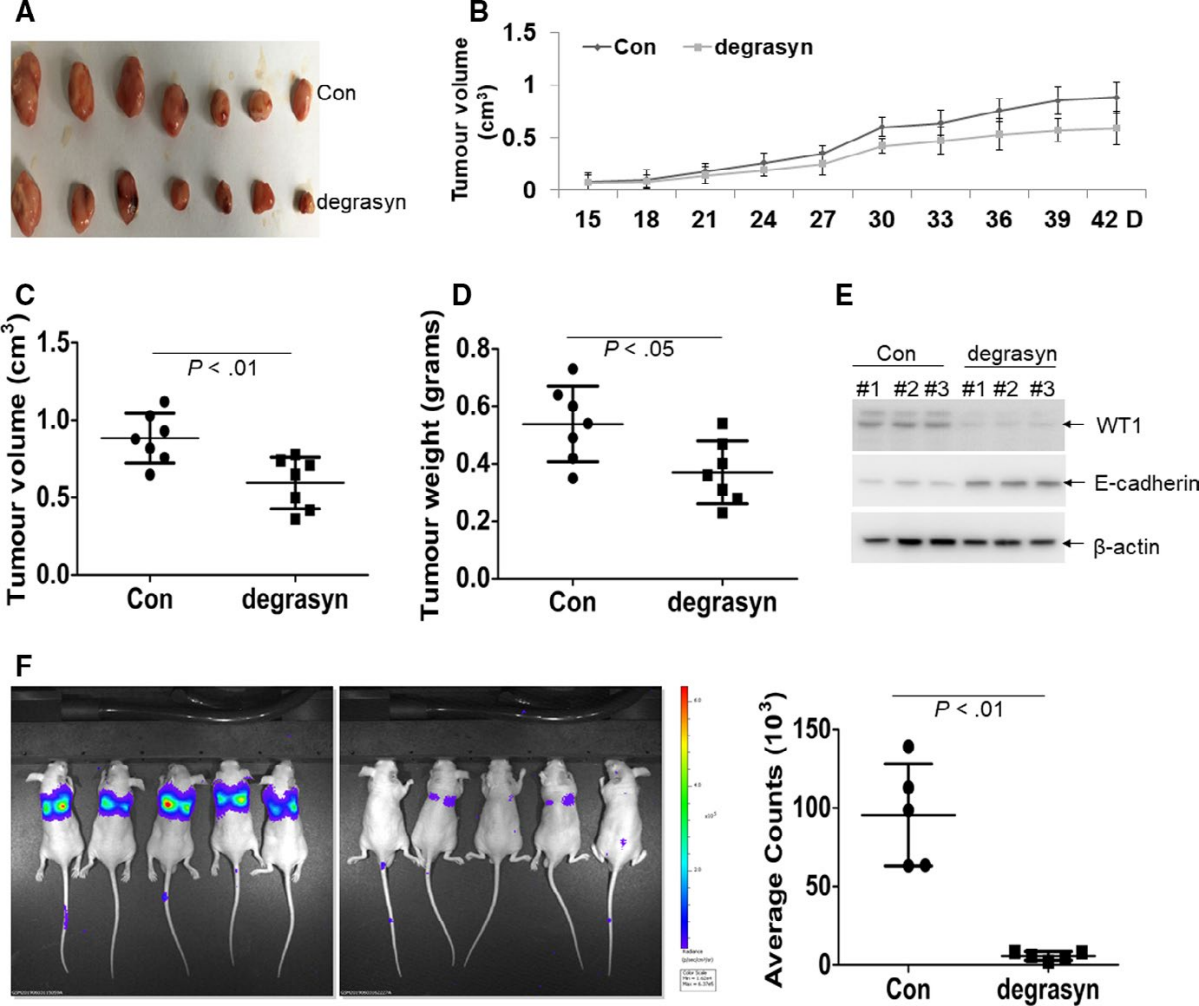
The anti‐cancer effects of degrasyn in PANC‐1‐xenografted mice model. (A) A photograph of tumours in degrasyn‐treated mice (N = 7) and control mice (N = 7). (B) Volumes of all tumours were detected every three days two weeks after tumour inoculation. (C) Volumes of all tumours were measured when the experiment was terminated at 42 days after tumour cell inoculation. (D) Net weights of all tumours were measured at the termination of the experiment. (E) The protein levels of WT1 and E‐cadherin were detected in three tumour lysates from degrasyn‐treated mice or control mice. (F) Luciferase intensity was performed in PANC‐1‐Luc‐transplanted mice, which were treated with or without degrasyn. Shown is the picture of the luciferase intensity (Left) and the summary of luciferase intensity (Right). N = 5 for each group

To determine whether degrasyn can reduce the tumorigenicity in a xenograft model, PANC‐1 cells were injected subcutaneously into right flank of mice to construct xenograft mouse model. All xenografted mice were divided in two groups according to degrasyn treatment or not. Tumours in degrasyn‐treated mice were significantly smaller than those in control mice (Figure 7A). Similarly, tumour growth was significantly reduced in degrasyn‐treated mice compared with control mice (Figure 7B). Furthermore, degrasyn reduced the average tumour volume by 43% compared with negative control (Figure 7C). Also, degrasyn treatment resulted in 36% decrease in average tumour weight (Figure 7D). Consistent with the results in cell lines, degrasyn reduced the protein levels of WT1 but increased E‐cadherin (Figure 7E).

Finally, PANC‐1 cells stably transduced with plenti‐Luciferase were injected into NSG mice from vein, establishing an animal model of metastasis. The role of anti‐metastasis by degrasyn was measured by a Caliper IVIS Lumina II system one month after tail vein injection. Degrasyn substantially reduced the fluorescence intensity than negative control (Figure 7F), indicating that degrasyn weakened the metastasis in PANC‐1‐transplanted mice model.

## 4 DISCUSSION

In this study, we investigated the anti‐cancer activity of deubiquitinase inhibitor degrasyn in pancreatic cancer. Our studies show that degrasyn suppresses proliferation, reduces colony and inhibits metastasis in pancreatic cancer cells through USP5‐WT1‐E‐cadherin axis. Moreover, we identified that zinc finger region is required for the degradation of WT1. Without degrasyn, overexpression of USP5 deubiquitinates WT1 protein and increases the expression of WT1, thus facilitating the metastasis through inhibiting E‐cadherin expression (Figure S4A). Treatment with degrasyn suppresses the activity of USP5, leading to the ubiquitination of WT1 and degradation of WT1 protein. The down‐regulation of WT1 inhibits the metastasis through increasing the expression of E‐cadherin (Figure S4B). Therefore, degrasyn might be a useful therapeutic agent for pancreatic cancer patients with high expressions of USP5 and WT1.

Targeting WT1 is a promising therapeutic strategy in various solid cancer because WT1 is an oncoprotein necessary for tumour growth and metastasis. As a transcript factor, WT1 maintains the survival and metastasis of cancer cells through modulating series of important genes. For example, WT1 increases the expressions of Bcl‐2^27^ and cyclin D1^9^ and down‐regulates the expression of E‐cadherin.^22^, ^28^ Thus, inhibiting WT1 is considered to indirectly regulate these important genes. Consistent with these reports, we found that inhibiting WT1 by shRNAs or degrasyn decreased the expression of WT1 followed by the up‐regulation of E‐cadherin in pancreatic cancer cells. Furthermore, small molecular compounds such as curcumin and histone deacetylase inhibitors present anti‐cancer activity by inhibiting WT1 expression through the PKCα signalling pathway^29^; histone deacetylase inhibitors like trichostatin A down‐regulates WT1 expression partially through ubiquitin‐conjugating enzyme UbcH8.^30^ These results indicate that WT1 might be a suitable target for cancer therapy. However, rapidly degrading WT1 protein by inhibiting deubiquitinase activity is not determined. Therefore, selecting effective agents which can rapidly degrade WT1 might provide a potentially attractive approach to cancer therapy.

Degrasyn is a small molecule that has a strong inhibition of deubiquitinating enzymes including USP5, USP14 and USP9x. Degrasyn decreases the expression of WT1 protein through inducing the accumulation of protein‐ubiquitin conjugates. For example, degrasyn induces proteasomal‐dependent degradation of c‐Myc in multiple myeloma.^19^ Moreover, degrasyn facilitates doxorubicin sensitivity through USP9x‐dependent degradation of p53 in hepatocellular carcinoma cells.^31^ Consistent with these reports, we found that degrasyn rapidly induces proteasomal‐dependent degradation of WT1 protein in pancreatic cancer cells. In addition to degrading oncoproteins through ubiquitin‐proteasome signalling pathway, degrasyn specifically and rapidly degrades both wild‐type and mutant Bcr‐Abl protein independent of ubiquitin‐proteasome pathway,^32^ suggesting a complicated role of degrasyn in eradicating oncoprotein. Overexpression of WT1 partially prevents degrasyn‐induced anti‐cancer activity, suggesting that degrasyn presents strong anti‐metastasis via degrading WT1 protein. Although curcumin inhibits pancreatic cancer cell proliferation via degrading WT1 protein,^12^ degrasyn decreases WT1 protein at lower concentration and presents more strong anti‐cancer activity than curcumin. Therefore, degrasyn might be a promising agent for treatment with pancreatic cancer patients through degrading oncoprotein WT1.

Deubiquitinating enzymes (DUBs) are a large group of ubiquitin‐specific proteases that can cleave ubiquitin from proteins and other molecules. Emerging reports indicate that many DUBs are differentially expressed or activated in different diseases, including various cancer, and might provide potential therapeutic targets. For example, USP9X stabilizes MCL‐1 and enhances tumour cell survival.^33^ USP7 deubiquitinates its substrate, the tumour suppressor CCDC6, and inhibiting USP7 activity determines CCDC6 degradation and sensitivity to PARP‐inhibitors treatment in tumour cells.^34^, ^35^ Here, we found that USP5 interacts with WT1 protein and stabilizes its expression. As documented, USP5 is significantly up‐regulated in PDAC cell lines and cancer tissues, leading to the enhanced tumorigenesis and progression of pancreatic cancer.^36^, ^37^ Thus, overexpression of USP5 might increase the expression of WT1 protein in PDAC cancer tissues. Inhibiting USP5 activity by degrasyn provides the potential therapeutic therapy in PDAC patients. However, further studies are still required to determine whether other ubiquitin‐specific proteases in addition to USP5 could deubiquitinate WT1 protein.

Almost 90% of pancreatic cancer patients express mutant KRAS (G12D), which has been considered as the initiator of PDAC.^38^ Although novel KRAS‐PDEdelta inhibitors seem to inhibit KRAS ability,^39^ the development of small molecule therapy targeting KRAS remains unsuccessful.^40^, ^41^ Thus, targeting the downstream of mutant KRAS is a candidate therapy for PDAC. WT1 has been reported to be a critical regulator of proliferation downstream of oncogenic KRAS signalling.^42^ Furthermore, WT1 loss decreases tumour burden in a mouse model of mutant KRAS‐driven cancer.^42^ Therefore, degrading WT1 protein by small molecule compounds such as degrasyn might inhibit oncogenic KRAS signalling.

USP5 has been well reported to cleave unanchored ubiquitin chains, replenishing the ubiquitin pool. Inhibiting USP5 activity by degrasyn might result in the decrease of ubiquitin pool. However, we speculate that this cleavage of unanchored ubiquitin chains might have little role in the observed effect of degrasyn, because ubiquitin is always sufficient for the ubiquitination of protein. Although USP5 has been reported to cleave unanchored ubiquitin chains, reports at present demonstrate that USP5 still can deubiquitinate protein‐conjugated ubiquitin chains. For example, USP5 was found to interact with c‐Maf and prevented it from degradation by decreasing its polyubiquitination level^43^; USP5 deubiquitinated β‐catenin^44^ and SLUG,^45^ finally leading to the increased expressions of β‐catenin and SLUG. Therefore, USP5 increases the expression of target protein via weakening the ubiquitination of target protein. Consistent with these reports, our data indicate that USP5 interacts with WT1 by Co‐IP experiments. Knockdown of USP5 enhances the ubiquitination of WT1 protein and overexpression of USP5 decreases the ubiquitination of WT1 protein using a ubiquitin‐binding assay. Furthermore, USP5 substantially decreased the ubiquitination of WT1 protein by a biochemical assay with purified USP5 in vitro. Therefore, our results demonstrate that USP5 might directly deubiquitinate WT1 protein.

Although WT1 is firstly considered as tumour suppressor,^5^ emerging studies demonstrate the oncogenic role of WT1 in leukaemia^46^ and solid cancer including lung cancer^9^ and pancreatic cancer.^6^ The complexity of the effect of WT1 overexpression and loss in cancer cell physiology is probably in part due to the fact that WT1 has multiple isoforms with distinct biological functions.^47^ While some isoforms of WT1 are able to bind DNA and act as transcription factors, other isoforms do not bind DNA but appear to participate in RNA processing.^48^ Therefore, discriminating the oncogenic or tumour suppress effect of WT1 might facilitate the understanding in the biological role of WT1 in PDAC. In this study, WT1 isoform A (Ex5‐KTS‐) was selected for the overexpression of exogenous WT1 to be degraded by degrasyn. Although WT1 isoform A can be degraded by degrasyn, further studies are required to determine whether other WT1 isoforms can be degraded by degrasyn.

In conclusion, our studies demonstrate that deubiquitinase inhibitor degrasyn possesses strong anti‐metastasis activity in vitro and in vivo through USP5‐mediated degradation of WT1. High expressions of USP5 and WT1 are associated with metastasis in PDAC patients. Thus, USP5 and WT1 should be potential target proteins for the preclinical treatment of PDAC patients. Gemcitabine is the first‐line drug for PDAC patients. However, gemcitabine treatment easily induces the drug resistance and following relapse.^49^ Combined treatment with gemcitabine and degrasyn might be performed in further preclinical trials to determine whether degrasyn could improve the clinical outcome in PDAC patients.

## CONFLICT OF INTERESTS

The authors declare that they have no competing interest.

## AUTHOR CONTRIBUTIONS

LJJ and LHY contributed to Western blot, migration assay, invasion assay, wound‐healing assay, qRT‐PCR, construction of plasmids, in vitro deubiquitination assay and virus package. ZWJ and ZB contributed to clinical samples collection, Co‐IP and Colony formation assay. YJC and WJS carried out RNA interference, mouse breeding and transplantation of pancreatic cancer cells. ZHX carried out IHC staining. SHW and GSM performed the study design, statistical analysis and manuscript writing. This manuscript is not under review elsewhere, and all authors read and approved the final manuscript.

## Data Availability

All data generated or analysed during this study are included in this article.
