# Supplementary material for: Deubiquitinase inhibitor degrasyn suppresses metastasis by targeting USP5‐WT1‐E‐cadherin signalling pathway in pancreatic ductal adenocarcinoma
Source: J Cell Mol Med. 2019 Dec 17;24(2):1370–82. doi: 10.1111/jcmm.14813 (PMC6991651; doi:10.1111/jcmm.14813)
Supplement: Supplementary file 1 [file JCMM-24-1370-s001.doc]

| Clinicopathologic variables | N | USP5^†^ | *P* value |
| --- | --- | --- | --- |
| All cases | 46 |  |  |
| Gender |  |  |  |
| Male | 32 | 144.6^‡^ (113.5-188.7) | 0.72 |
| Female | 14 | 136.7 (108.3-195.2) |  |
| Age (years) |  |  |  |
| <60 | 13 | 142.1 (98.4-196.3) | 0.31 |
| ≥60 | 33 | 140.4 (103.1-194.0) |  |
| Primary tumor |  |  |  |
| T1/2 | 14 | 145.7 (102.0-189.7) | 0.15 |
| T3/T4 | 32 | 153.2 (105.4-198.2) |  |
| Regional lymph nodes |  |  |  |
| N0 | 31 | 127.3 (94.2-175.6) | 0.23 |
| N1 | 15 | 135.8 (99.5-181.2) |  |
| ^§^Distant metastasis |  |  |  |
| M0 | 18 | 110.4 (80.1-162.6) | **0.02*** |
| M1 | 28 | 162.3 (143.7-220.3) |  |
| Differentiation |  |  |  |
| Well or moderately | 16 | 126.7 (86.9-181.3) | 0.56 |
| Poorly or none | 30 | 124.9 (91.5-173.4) |  |

**Table S1: Clinicopathologic features and USP5 expression in 46 PDAC patients**

**P*<0.05 (shown in bold).

^†^USP5 expression is shown as protein integrated optical density.

^‡^median of USP5 relative expression and the 25th–75th percentiles of USP5 expressions are listed in parentheses.

^§^According to UICC/AJCC Guideline Version 7 Pancreatic Cancer staging.

Abbreviation: USP5: Ubiquitin-specific protease 5; PDAC: Pancreatic ductal adenocarcinoma
