# Supplementary material for: Deubiquitinase inhibitor degrasyn suppresses metastasis by targeting USP5‐WT1‐E‐cadherin signalling pathway in pancreatic ductal adenocarcinoma
Source: J Cell Mol Med. 2019 Dec 17;24(2):1370–82. doi: 10.1111/jcmm.14813 (PMC6991651; doi:10.1111/jcmm.14813)
Supplement: Supplementary file 2 [file JCMM-24-1370-s002.doc]

| Clinicopathologic variables | N | WT1^†^ | *P* value |
| --- | --- | --- | --- |
| All cases | 46 |  |  |
| Gender |  |  |  |
| Male | 32 | ^‡^ 98.4 (46.1-146.7) | 0.57 |
| Female | 14 | 95.3 (51.6-163) |  |
| Age (years) |  |  |  |
| <60 | 13 | 101.1 (51.6-159.1) | 0.68 |
| ≥60 | 33 | 104.9 (48.3-173) |  |
| Primary tumor |  |  |  |
| T1/2 | 14 | 106.2 (62.8-156.2) | 0.29 |
| T3/T4 | 32 | 112.5 (50.9-166.4) |  |
| Regional lymph nodes |  |  |  |
| N0 | 31 | 108.9 (53.2-158.3) | 0.52 |
| N1 | 15 | 110.5 (55.8-152.6) |  |
| ^§^Distant metastasis |  |  |  |
| M0 | 18 | 88.3 (67.8-123.0) | ***0.03** |
| M1 | 28 | 132.6 (94.4-172.8) |  |
| Differentiation |  |  |  |
| Well or moderately | 16 | 101.7 (58.2-163.4) | 0.34 |
| Poorly or none | 30 | 112.8 (52.7-167.5) |  |

**Table S2: Clinicopathologic features and WT1 expression in 46 PDAC patients**

**P*<0.05 (shown in bold).

^†^WT1 expression is shown as protein integrated optical density.

^‡^median of WT1 relative expression and the 25th–75th percentiles of WT1 expressions are listed in parentheses.

^§^According to UICC/AJCC Guideline Version 7 Pancreatic Cancer staging.

Abbreviation: WT1: Wilm’s tumor-1; PDAC: Pancreatic ductal adenocarcinoma
