# Supplementary material for: Deubiquitinase inhibitor degrasyn suppresses metastasis by targeting USP5‐WT1‐E‐cadherin signalling pathway in pancreatic ductal adenocarcinoma
Source: J Cell Mol Med. 2019 Dec 17;24(2):1370–82. doi: 10.1111/jcmm.14813 (PMC6991651; doi:10.1111/jcmm.14813)
Supplement: Supplementary file 3 [file JCMM-24-1370-s003.doc]

| Gene | Sequences |
| --- | --- |
| WT1-L | 5′-CAA TCA GGG TTA CAG CAC GG-3′ |
| WT1-R | 5′-GCT TGA ATG AGT GGT TGG GG-3′ |
| USP5-L | 5′-GCT GCT GTC AGT ATT ACC GAC-3′ |
| USP5-R | 5′- AAA GCC CAG AAA CGT GTT CAT A-3′ |
| β-actin-L | 5′-TGG CAT CCA CGA AAC TAC CT-3′ |
| β-actin--R | 5′-CGT ACA GGT CTT TGC GGA TG-3′ |
| LVX-USP5-L | 5′-CCG CTC GAG ATG GCG GAG CTG AGT GAG GA-3′ |
| LVX-USP5-R | 5′-GCT CTA GAG CTT AGC TGG CCA CTC TCT GGT-3′ |
| MSCV-WT1-L | 5′-GAA GAT CTC TGC AGG ACC CGG CTT CCA C-3′ |
| MSCV-WT1-R | 5′-CCG GAA TTC TCA AAG CGC CAG CTG GAG TT-3′ |
| pCMV-WT1-L | 5′-CGG AAT TCA CTG CAG GAC CCG GCT TCC-3′ |
| pCMV-WT1-R | 5′-CCC AAG CTT TCA AAG CGC CAG CTG GAG-3′ |
| sh-WT1#1 | 5′-GCA GTG ACA ATT TAT ACC AAA-3′ |
| sh-WT1#2 | 5′-GAT GAA CTT AGG AGC CAC CTT-3′ |
| sh-USP5#1 | 5′-CGG GCC ACG AAC AAT AGT TTA-3′ |
| sh-USP5#2 | 5′-AGC GAG GAG AAG TTT GAA TTA-3′ |
| sh-USP9x#1 | 5′-GGT CGT TAC AGC TAG TAT TTA -3′ |
| sh-USP9x#2 | 5′-CGA CCC TAA ACG TAG ACA TTA-3′ |
| sh-USP14#1 | 5′-GCA GCC CTT AGA GAT TTG TTT-3′ |
| sh-USP14#2 | 5′-GCA GCC AAA TAC AAG TGA CAA-3′ |

**Table S3: The sequences of primers for qRT-PCR and construction of plasmids**
