# Supplementary figures and images for: Deubiquitinase inhibitor degrasyn suppresses metastasis by targeting USP5‐WT1‐E‐cadherin signalling pathway in pancreatic ductal adenocarcinoma
Source: J Cell Mol Med. 2019 Dec 17;24(2):1370–82. doi: 10.1111/jcmm.14813 (PMC6991651; doi:10.1111/jcmm.14813)

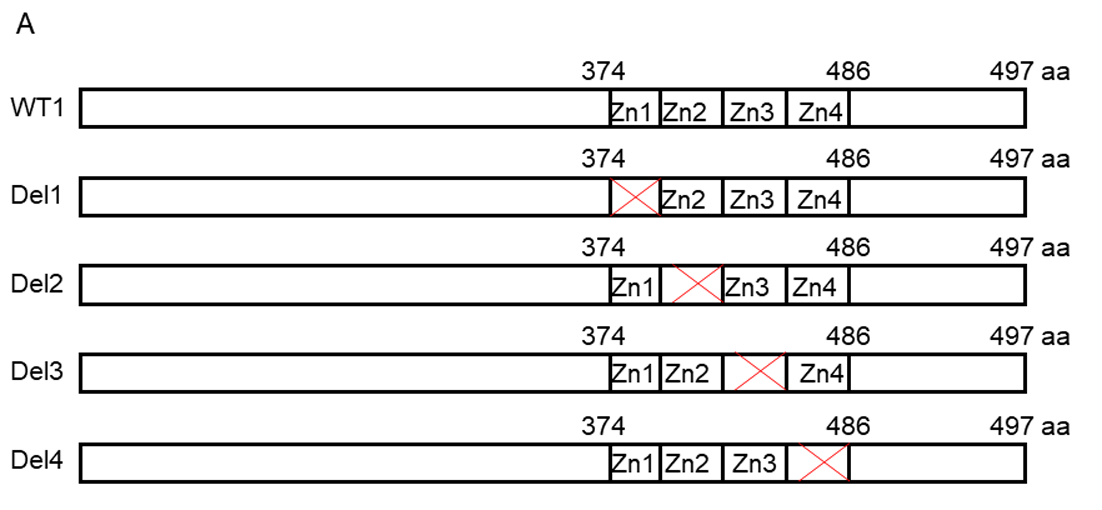

Supplement: Supplementary file 4 [file JCMM-24-1370-s004.tif]

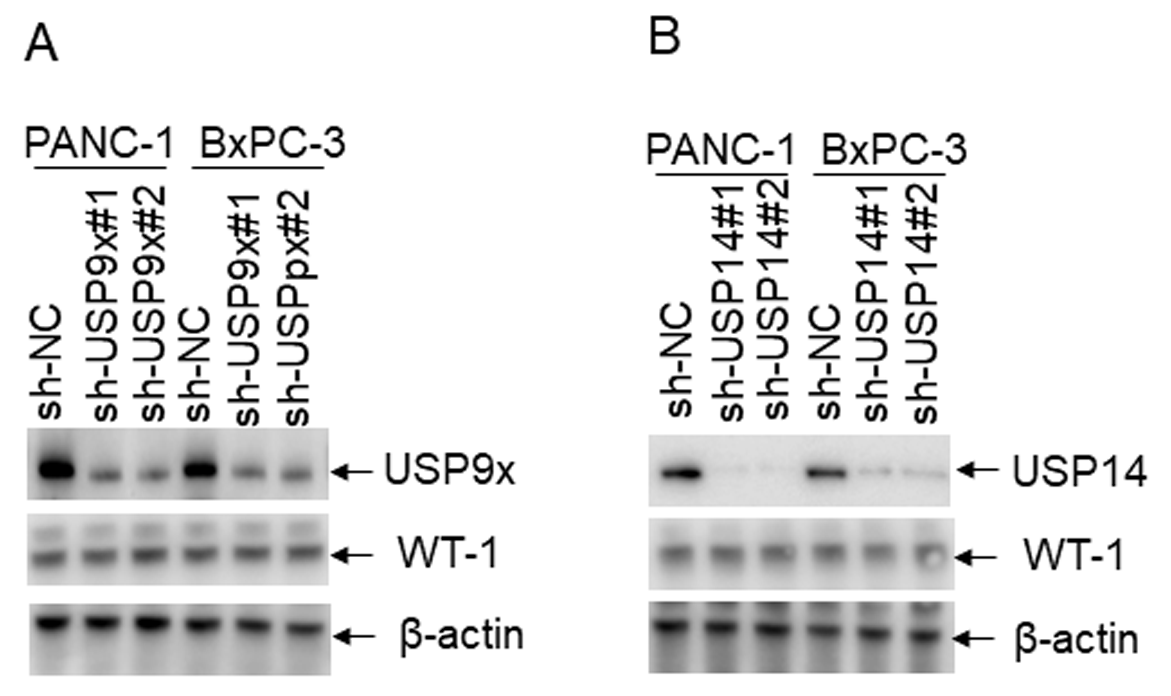

Supplement: Supplementary file 5 [file JCMM-24-1370-s005.tif]

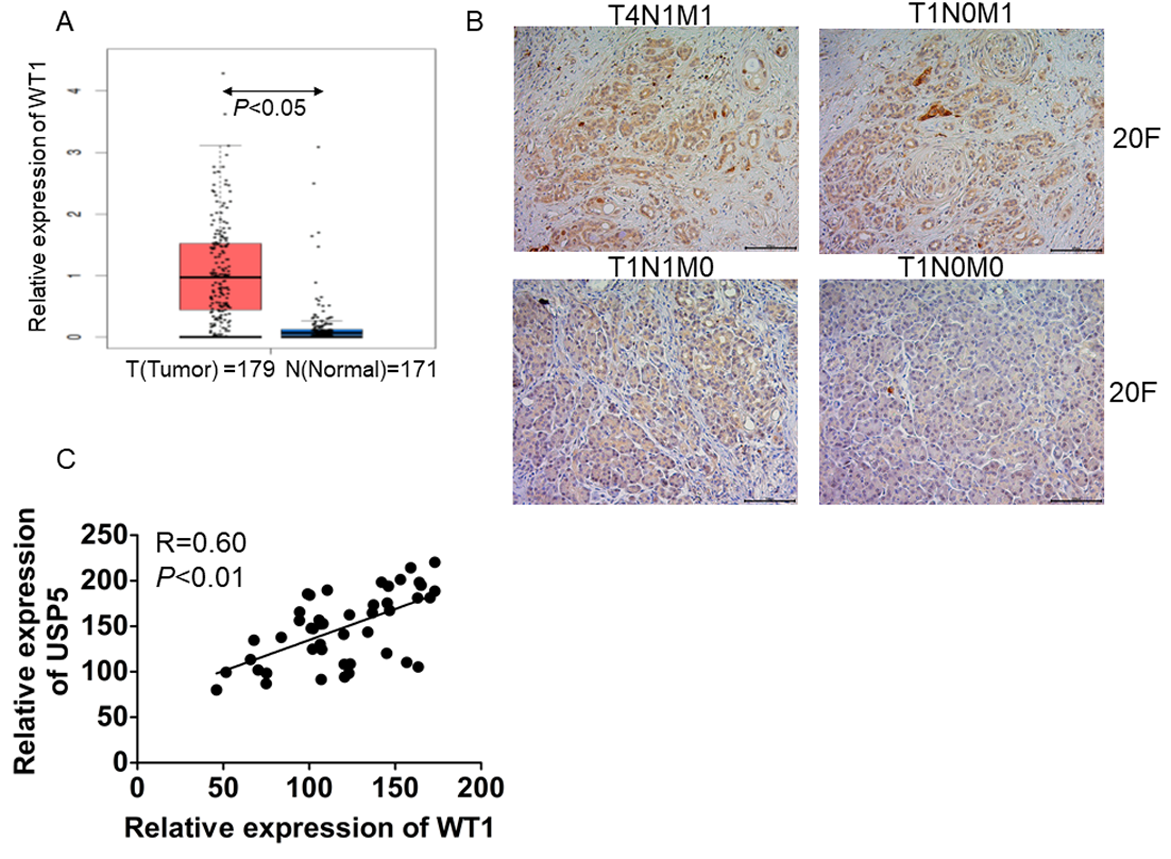

Supplement: Supplementary file 6 [file JCMM-24-1370-s006.tif]

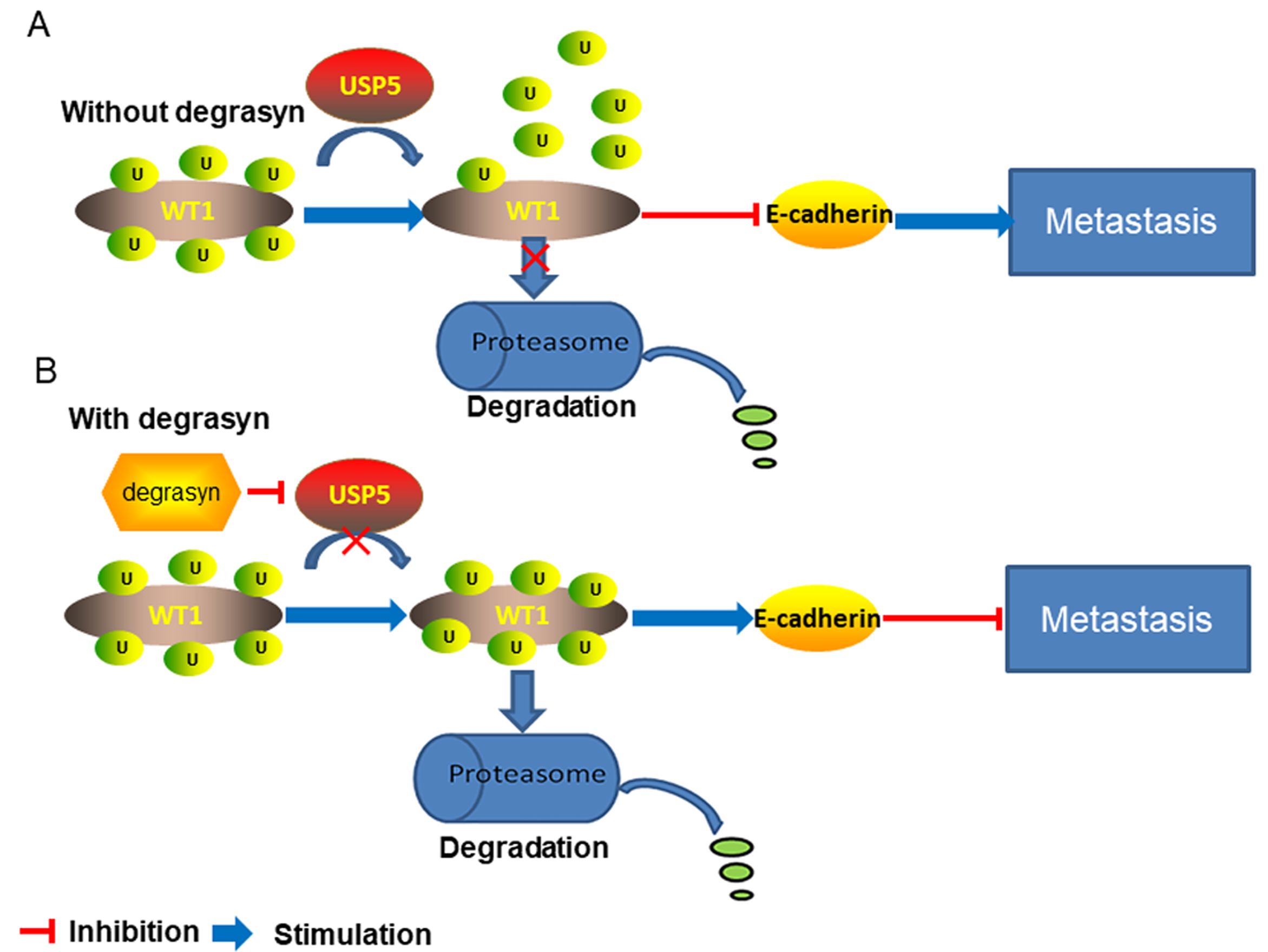

Supplement: Supplementary file 7 [file JCMM-24-1370-s007.tif]
