# Supplementary material for: Deubiquitinase inhibitor degrasyn suppresses metastasis by targeting USP5‐WT1‐E‐cadherin signalling pathway in pancreatic ductal adenocarcinoma
Source: J Cell Mol Med. 2019 Dec 17;24(2):1370–82. doi: 10.1111/jcmm.14813 (PMC6991651; doi:10.1111/jcmm.14813)
Supplement: Supplementary file 8 [file JCMM-24-1370-s008.docx]

Supplemental Figure Legend

Fig S1. Zinc finger region is involved in its sensitivity to degrasyn. (A) Representation of wide-type WT1 protein and deletion of every zinc finger.

Fig S2. Knockdown of USP9x and USP14 does not modulate the expression of WT1 in PANC-1 and BxPC-3 cells. (A) The protein expressions of USP9x and WT1 were detected in PANC-1 and BxPC-3 cells, which were transduced with shRNAs for USP9x or sh-NC. (B) PANC-1 and BxPC-3 cells were transduced with shRNAs for USP14 or sh-NC, followed by western blot for USP14 and WT1.

Fig S3. WT1 is associated with metastasis in PDAC patients. (A) The mRNA expression of *WT1* was analyzed in Genotype-Tissue Expression (GTEx) portal database and TCGA database. (B) Representative immunohistochemical staining for WT1 in PDAC tissues with different tumor stages. (C) WT1 and USP5 expressions were measured by IHC staining in 46 PDAC tissues and the correlation coefficient (R) and *P* values were detected by Pearson correlation.

Fig S4. An illustration of USP5-mediated degradation of WT1 protein by degrasyn. (A) Without degrasyn, overexpression of USP5 maintains the high expression of WT1 through deubiquitinating WT1 protein and preventing it from degradation by ubiquitin-proteasome signaling pathway, thus contributing to the metastasis through inhibiting E-cadherin in pancreatic cancer cells. (B) After degrasyn treatment, USP5 activity is inhibited by degrasyn, leading to the degradation of WT1 protein through ubiquitin-proteasome signaling pathway. The downregulated expression of WT1 increases the level of E-cadherin, finally inhibiting the metastasis in pancreatic cancer cells.
