# Supplementary material for: Deubiquitinase inhibitor degrasyn suppresses metastasis by targeting USP5‐WT1‐E‐cadherin signalling pathway in pancreatic ductal adenocarcinoma
Source: J Cell Mol Med. 2019 Dec 17;24(2):1370–82. doi: 10.1111/jcmm.14813 (PMC6991651; doi:10.1111/jcmm.14813)
Supplement: Supplementary file 9 [file JCMM-24-1370-s009.docx]

**SUPPLEMENTAL MATERIAL AND METHODS**

**2.1 RNA interference**

To produce the plasmids carrying gene-specific short hairpin RNA (shRNA) for *WT1*, *USP9x*, *USP14*, and *USP5*, target sequences were designed and cloned into pSIREN-RetroQ (Clontech) retroviral vector, respectively. Control shRNA is a nonfunctional construct provided from Clontech. The sequences of shRNAs were indicated in Table S3. These vectors expressing shRNAs as well as negative control vector were co-transfected with packaging plasmids (Gap-pol and VSV-G) into 293T cells to produce retrovirus. Supernatants containing retrovirus were collected and filtered by 0.45 μM filter after transfection for 48 h. Pancreatic cancer cells were transduced with retrovirus and positive clones with stable knockdown of *WT1*, *USP9x*, *USP14*, and *USP5* were selected by puromycin (Selleckchem) for one week.

**2.2 Retrovirus and lentivirus production and cell transduction**

HEK293T cells (4×10^6^) were plated in 10 cm dish. After 24 h, MSCV-based vectors and pLVX-based vectors were cotransfected with packaging plasmids into HEK293T cells. The supernatant containing virus was harvested at 48 h after transfection. PANC-1 and BxPC-3 were cultured in 6-well plate for 24 h before transduction. Virus mixed with polybrene (8 µg/ml, Sigma-Aldrich, St. Louis, USA) were added in 6-well plate. Positive clones with overexpression of WT1 and USP5 were selected by 1 µg/ml puromycin (Selleckchem) treatment for one week.

**2.3 RNA extraction and qRT-PCR**

Total RNA was extracted using Trizol reagent (Invitrogen, Carlsbad, CA, USA) following the standard procedure. Briefly, pancreatic cancer cells were lysed with 1 ml Trizol followed by intense mixture for five min at room temperature. The lysates were vigorously mixed with chloroform (200 μl) and laid for 15 mins at room temperature. After centrifugation for 15 mins at 4 °C, upper solution was extracted and isopropanol (500 μl) was applied to precipitate RNA. Finally, RNA was dried and dissolved with RNase-free water. RNA concentration and quality were measured by the absorbance at 260 nm using Beckman DU6400 spectrophotometer (Beckman Counter, Miami, FL, USA). Real-time PCR was performed using SYBR Green PCR Master Mix (Qiagen) in an Applied Biosystems 7900 instrument. Expression data were uniformly normalized to the internal control and the relative expression levels were evaluated using the 2^-ΔΔCt^ method. The primer sequences were indicated in Table S1.

**2.4 Co-****immunoprecipitation (co-IP) assays**

To identify the interaction between endogenous WT1 and USP5, WT1 and USP5 were immunoprecipitated by anti-USP5 (1:200) or anti-WT1 antibody (1:200), respectively, using the Pierce™ coimmunoprecipitation kit (#88804, Thermo Scientific) according to the manufacturer’s protocol. Briefly, PANC-1 cells were lysed in cold RIPA lysis buffer. After centrifugation, the supernatant was transferred to new tubes and 50% protein A/G agarose was added to eliminate non-specific binding proteins. Appropriate amount of primary antibody was added in the tube and slowly shake antigen-antibody complex on rotating shaker at 4°C for overnight. After binding antigen/antibody complex to protein A/G magnetic beads for 1 hour at RT, the beads were washed with IP lysis and wash buffer. The antigen/antibody complex was eluted for the following SDS-PAGE.

**2.5 Tumorigenicity in nude mice**

Male athymic nude mice (6-week-old) were purchased from SLAC (Shanghai SLAC Laboratory Animal, Shanghai, China) and were housed in blanket cages with food and water available. All animal procedures and care were conducted in compliance with national and international laws and policies and in accordance with institutional guidelines of the First Affiliated Hospital of Wenzhou Medical University. All surgery was performed under sodium pentobarbital anesthesia, and all efforts were made to minimize suffering. To produce xenograft model for WT1 knockdown, viable PANC-1 cells with sh-NC (1х10^7^) or sh-WT1#1 (1х10^7^) were trypsinized and resuspended with 100 μl sterile 1×PBS mixed with high concentration of MatrigelTM Matrix (1:1 ratio, BD Bioscience, San Jose, CA, USA). These cells were injected subcutaneously into right flank of each nude mouse. To construct degrasyn-treated xenograft model in nude mice, viable 1х10^7^ PANC-1 cells were trypsinized and resuspended with 100 μl sterile 1×PBS mixed with high concentration of MatrigelTM Matrix (1:1 ratio, BD Bioscience) and were injected subcutaneously into right flank of each nude mouse. Mice were randomly divided into two groups after inoculation for three weeks. One group of mice (N=7) was received intraperitoneal injections with degrasyn (25 mg/kg) dissolved in PEG3000/DMSO (1:1) once every other day until termination. The other group of mice (N=7) was received an equal volume of PEG3000/DMSO serving as vehicle group. Mice were sacrificed and the tumors from each mouse were harvested for measuring volume and weight when the experiment was terminated at six weeks after tumor cell inoculation. Tumor volumes were measured using the equation V (in cm^3^) = AхB^2^/2, where A is the largest diameter and B is the perpendicular diameter.

**2.6 Tumor metastasis assay *in vivo*.**

PANC-1 cells (2×10^6^) transduced with pLenti-Luciferase were resuspended in 100 μL PBS and injected into the tail vein of 6-week-old male athymic nude mice under specific pathogen-free conditions. After two weeks’ inoculation, mice were randomly divided into two groups: degrasyn (25 mg/kg)-treated mice (N=5) and vehicle mice (N=5). Mice were intraperitoneally injected with degrasyn dissolved in PEG3000/DMSO (1:1) once every other day until termination. Vehicle mice were received an equal volume of PEG3000/DMSO. Tumor metastasis was measured by an IVIS Lumina X5 system (PerkinElmer Health Sciences, Waltham, MA, USA) and analyzed by Living Image software (PerkinElmer Health Sciences).

**2.7 In vitro deubiquitination assay**

To determine whether USP5 directly de-ubiquitinates WT1, Flag-tagged WT1 was coexpressed with HA-tagged ubiquitin (HA-Ub) in 293T cells and then was purified by an anti-Flag antibody. The protein was purified by Protein A/G Plus agarose beads (Thermo Scientific) under denaturing condition (10 mM DTT, 1 mM EDTA, 50 mM Tris-HCl, 50 mM NaCl, and 5% glycerol). Then, ubiquitinated-WT1 proteins were incubated with purified USP5 protein (SinoBiological, Beijing, China) in deubiquitination buffer at 37 °C for 4 hours. The reaction was terminated by boiling in 2×SDS sample buffer for 10 min. Then, the samples were resolved on SDS-PAGE gels and measured by western blot analysis.
